# Spontaneous Bilateral Subdural Hematomas Associated With Resolving Hypertensive Intraparenchymal Hemorrhage: A Case Report

**DOI:** 10.7759/cureus.88851

**Published:** 2025-07-27

**Authors:** Joshua Martin Manogaran, Solangaratchige Don Harshana Sameera Perera, Faizah Lubna, Vetha Irene Sanjana

**Affiliations:** 1 Department of Stroke Medicine, Hillingdon Hospital, London, GBR; 2 Department of Internal Medicine, Hillingdon Hospital, London, GBR

**Keywords:** acute hemorrhagic stroke, bilateral subdural hemorrhage, intracerebral hematoma, intracranial hematoma resolution, intracranial hypo-tension, intraparenchymal hematoma, low intracranial pressure, spontaneous subdural hemorrhage, subdural hemorrhage, subdural hemorrhage causes

## Abstract

Intraparenchymal hemorrhage (IPH) and subdural hematoma (SDH) are well-recognized separate clinical entities. We present a case of spontaneous bilateral SDHs identified during the resolution phase of a hypertensive IPH in a 73-year-old male. The patient initially presented unconscious with an acute right-sided thalamoganglionic IPH confirmed via non-contrast brain computed tomography scan. Conservative management led to neurological improvement, but routine follow-up imaging at eight weeks unexpectedly demonstrated bilateral SDHs without clinical deterioration. Further imaging at 14 weeks indicated progressive enlargement of the right-sided SDH. Extensive diagnostic evaluations, including magnetic resonance imaging and magnetic resonance venography, identified no underlying vascular or structural abnormalities, besides ruling out other predisposing factors such as coagulation disorders, preceding trauma, or brain atrophy. We hypothesize that intracranial pressure fluctuations during the resolution of IPH contribute to the formation of spontaneous SDH. This case highlights a potential cause of unexplained SDH in the context of a resolving IPH, underscoring the knowledge gap in pathophysiology and its underlying mechanisms.

## Introduction

Intraparenchymal hemorrhage (IPH) is characterized by bleeding into the brain parenchyma. Despite significant advances in diagnostic imaging, blood pressure control, and understanding of the pathophysiology of IPH, a recent study showed that the incidence of IPH has remained stable over the last four decades [1]. This stagnation raises concerns about unidentified risk factors, underutilization of preventive strategies, and the complexity of IPH pathogenesis.

IPH can result from both traumatic and non-traumatic causes. Non-traumatic IPH is subdivided into primary and secondary etiologies. Primary IPH, accounting for up to 85% of spontaneous hemorrhages, is related to chronic hypertension and cerebral amyloid angiopathy (CAA) [2,3]. Chronic hypertension induces degenerative changes in small penetrating arteries and formation of Charcot-Bouchard microaneurysms, which predispose to deep cerebral bleeds, particularly in the basal ganglia, thalamus, and brainstem [3]. Diagnosing hypertensive IPH requires exclusion of structural, vascular, neoplastic, or coagulopathic causes through neuroimaging, blood investigations, and clinical correlation, including assessment of hypertensive end-organ damage.

CAA is another primary etiology that typically presents with lobar hemorrhages. It results from amyloid-β deposition in the cortical and leptomeningeal vessels, leading to vessel fragility [4]. Diagnosis is supported by clinical presentation and imaging features such as cerebral microbleeds, and by application of the Boston criteria [5]. Secondary causes of IPH include arteriovenous malformations, vascular aneurysms, hemorrhagic transformation of infarct, tumors, coagulopathies, and iatrogenic factors [2].

Subdural hematoma (SDH) is a distinct condition caused by the collection of blood between the dura mater and arachnoid mater of the brain. While spontaneous/non-traumatic SDH has been described in association with coagulation disorders, vascular lesions, and intracranial hypotension, its development in the context of resolving IPH is uncommon. To our knowledge, bilateral SDHs arising during the resolution of a hypertensive IPH without any traumatic and clinical deterioration have not been reported in the literature. This case report aims to explore this uncommon association and propose a possible explanation through fluctuations in intracranial pressure (ICP), highlighting the diagnostic uncertainty and clinical significance it may carry.

## Case presentation

A 73-year-old man with a medical history of hypertension, epilepsy (no documented seizure for more than 12 years), and hypercholesterolemia was brought to the emergency department of a district general hospital after an unwitnessed collapse. Upon arrival, his Glasgow Coma Scale (GCS) score was 6/15 (E1 V1 M4), necessitating intubation to protect the airway. On examination, there was no movement of the left upper and lower limbs in response to pain, while a withdrawal response was observed to a painful stimulus on the right side. He was hemodynamically stable, with a blood pressure of 145/75 mmHg. There were no signs of external trauma. He had no significant family history. Regular medications included Amlodipine 10 mg, Levetiracetam 250 mg, and Simvastatin 20 mg; however, compliance with the medications was uncertain. His baseline was being independent with all daily activities and no risk factors, including smoking or alcohol consumption. 

**Figure 1:**
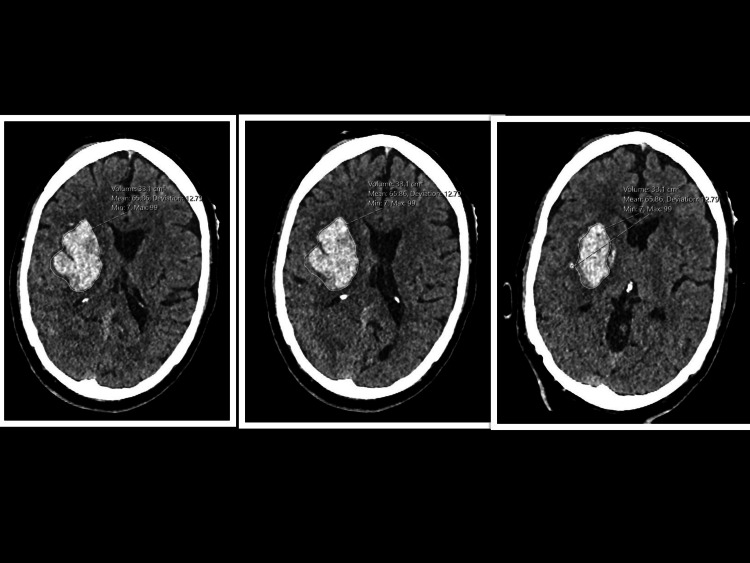
Non-contrast axial CT scan of the brain on presentation (Day 1). An acute right thalamoganglionic hemorrhage measuring approximately 33.1 cm³, with partial effacement of the overlying convexity sulci and adjacent ventricle, and a 5-6 mm leftward midline shift.

The initial non-contrast computed tomography (CT) of the brain showed an acute right-sided thalamoganglionic hemorrhage of volume 33.1 cm^3^, accompanied by mild surrounding edema (Figure 1). CT angiography and CT of the cervical spine identified no underlying vascular malformation but showed a stable C5 vertebral fracture. The initial working diagnosis was a hypertensive deep cerebral hemorrhage. Following this, the patient was admitted to the intensive care unit.

Laboratory investigations, including a complete blood count, coagulation profile, renal and liver function tests, lipid panel, and autoimmune screens, were all within normal limits (Table 1). These were performed to rule out coagulopathy and vasculitis as possible secondary causes of intracerebral hemorrhage. Lumbar puncture was not indicated, as there were no features of meningitis or encephalitis, and there were no clinical signs to suggest other infections or ongoing seizure activities necessitating an electroencephalogram. 

**Table 1:** Blood test results on initial presentation. ANCA, anti-neutrophil cytoplasmic antibodies; HDL, high-density lipoprotein; LDL, low-density lipoprotein

Laboratory test	Value	Normal range	Units
White blood cell	10.6	4.2-10.6	x10^9/L
Neutrophil count	6.8	2-7.1	x10^9/L
Lymphocytes	1.6	1.1-3.6	x10^9/L
Hemoglobin	126	130-168	g/L
Hematocrit	0.379	0.39-0.50	L/L
Mean cell volume	93.1	83.9-99.5	fL
Platelet count	229	130-370	x10^9/L
Fibrinogen	2.84	1.9-4.3	g/L
Prothrombin time	14.1	12.8-17.4	seconds
International normalized ratio	1	0.8-1.2	ratio
Activated partial thromboplastin time	28.1	23.9-35.5	seconds
Sodium	138	133-146	mmol/L
Potassium	3.6	3.5-5.3	mmol/L
Chloride	104	95-108	mmol/L
Magnesium	0.85	0.7-1.0	mmol/L
Creatinine	48	64-104	µmol/L
Urea	3.3	2.5-7.8	mmol/L
Estimated glomerular filtration rate	>90	>90	ml/min/1.73m^2
Alanine aminotransferase	66	0-45	unit/L
Alkaline phosphatase	127	30-130	unit/L
Total protein	64	60-80	g/L
Albumin	38	35-50	g/L
Globulin	26	19-35	g/L
Total bilirubin	7	0-21	µmol/L
Adjusted calcium	2.06	2.2-2.6	mmol/L
Amylase	37	0-100	unit/L
C-reactive protein	18	<5.0	mg/L
Complement C3	1.49	0.82-1.85	g/L
Complement C4	0.16	0.15-0.53	g/L
Rheumatoid factor	<20	0-29	IU/mL
Antinuclear antibody	Negative	1:40	ratio
ANCA proteinase 3 antibody	Negative	<2	IU/mL
ANCA myeloperoxidase antibody	Negative	<3.5	IU/mL
Cardiolipin immunoglobulin G antibody	Negative	<20	GPLU/mL
Cardiolipin immunoglobulin M antibody	Negative	<20	GPLU/mL
Anti-beta 2 glycoprotein 1 immunoglobulin M	Negative	<20	U/mL
Anti-beta 2 glycoprotein 1 immunoglobulin G	Negative	<20	U/mL
Human immunodeficiency viruses 1 and 2 serology	Not detected	-	-
Cholesterol level	3.9	<2.5	mmol/L
Triglyceride level	1.1	<1.7	mmol/L
HDL cholesterol level	1.2	>1.0	mmol/L
LDL cholesterol level	2.2	<2.6	mmol/L
Total cholesterol:HDL ratio	3.3	<5	ratio

The neurosurgical team advised non-operative management, given the deep location of the hemorrhage and the absence of a significant midline shift, while closely monitoring the GCS score for any further decline. The patient was managed conservatively, including blood pressure control. The C5 vertebral fracture was managed with a cervical collar. The regular antiepileptic medications were continued, given the history of epilepsy. 

Over the following two weeks, the patient showed gradual neurological improvement, as evidenced by a GCS improving to 13. He was extubated and transferred to the stroke rehabilitation unit. On examination, he demonstrated expressive aphasia and left-sided dense hemiplegia. Functional recovery continued under a multidisciplinary rehabilitation program, with a Modified Rankin Scale (mRS) score of 4. There were no recurrent seizures or new complications.

**Figure 2:**
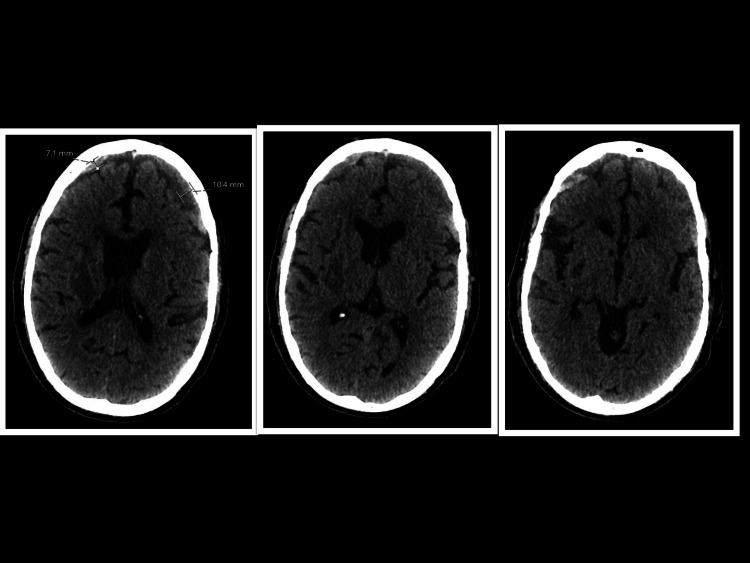
Non-contrast axial CT scan of the brain - week 8 Bilateral subdural hematomas (SDH) over the frontal lobes, right measuring 7.1 mm and left measuring 10.4 mm, with varying intensities, predominantly isodense - on a background of resolving right intraparenchymal hemorrhage (IPH).

A routine follow-up CT brain at eight weeks revealed new bilateral subdural hematomas, more prominent on the left, in the context of resolving IPH (Figure 2). The patient had no history of falls or trauma during admission. Given his stable GCS score, absence of symptoms such as headache, and lack of new or worsening focal neurological deficits, conservative management was continued.

**Figure 3:**
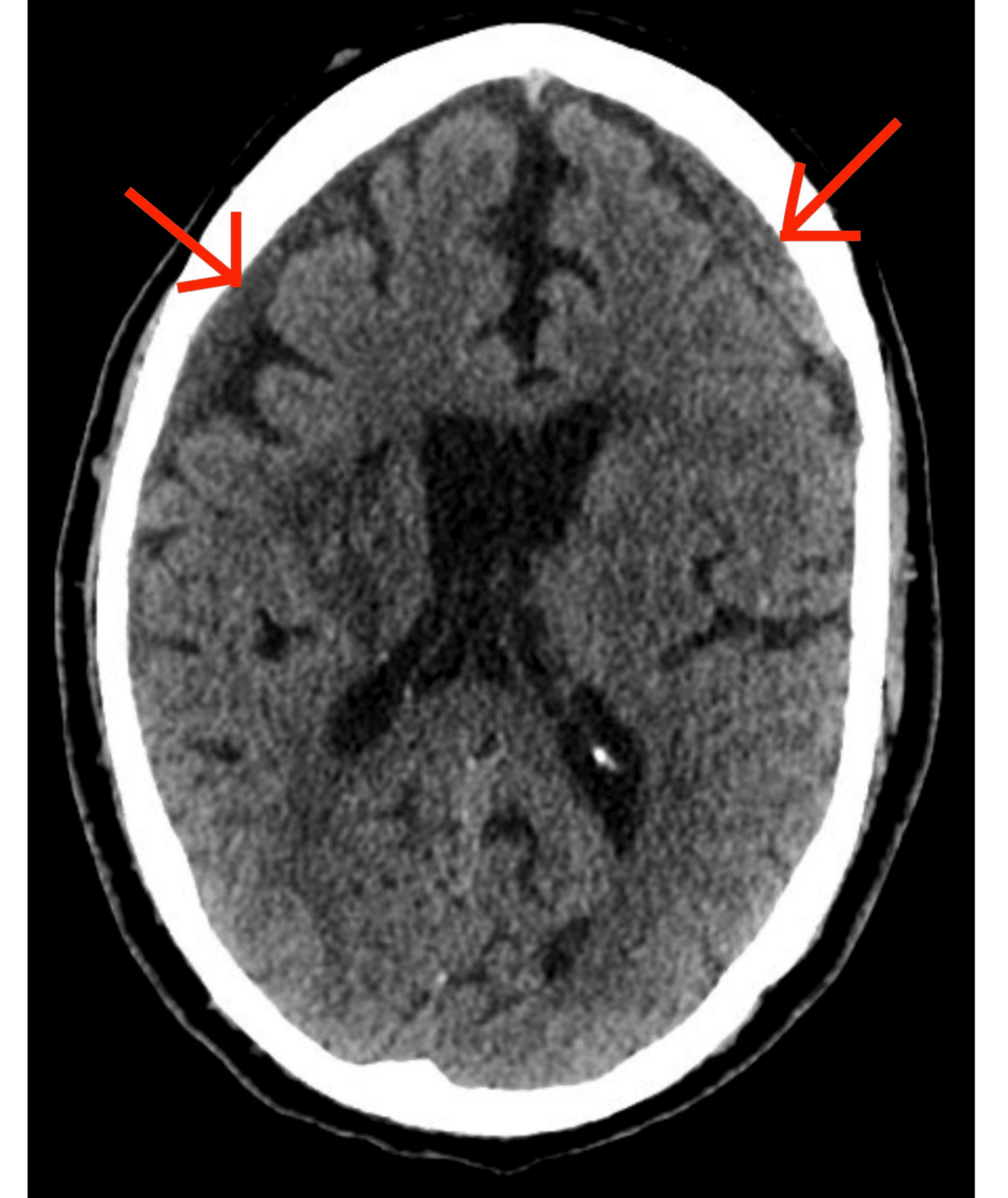
Non-contrast CT scan (axial) - brain - week 14. Bilateral subdural hematomas (SDH), with a stable left SDH and an expanding right SDH, on a background of resolving right intraparenchymal hemorrhage (IPH). Varying intensities are seen in the left-sided SDH, ranging from hypodense to a few areas of hyperdensity.

A subsequent 14-week non-contrast CT scan showed mild interval expansion of the right-sided SDH (Figure 3) with no midline shift, again in the absence of clinical deterioration or new symptoms. There were no new neurological deficits or signs of raised intracranial pressure, such as headache, vomiting, or papilledema. MRI and MR venography of the brain were performed to evaluate for vascular abnormalities or other secondary causes, such as malignant lesions, but no abnormalities were identified. 

The patient continued a rehabilitation program over the next eight weeks, showing gradual progression to an mRS score of 3. There was no new change in the clinical picture, including headache, vomiting, or focal neurological symptoms and signs. He was discharged with safety netting with warning signs, including headache or new focal neurological symptoms. This unexplained formation of bilateral SDH and expansion in the absence of symptomatology led to a diagnostic dilemma and uncertainty of clinical significance.

## Discussion

This case highlights an uncommon occurrence of bilateral spontaneous SDHs developing in the resolution phase of a hypertensive IPH, without any evidence of trauma, coagulopathy, or vascular malformation or risks such as brain atrophy or previous SDH. The lack of an identifiable cause for the SDHs, despite a comprehensive diagnostic workup and the absence of clinical deterioration, raises important questions about the pathophysiological mechanisms involved and clinical significance.

Spontaneous SDH in the setting of resolving IPH is not well described in the literature. In this case, no clear precipitant, such as coagulation disorder, recent lumbar puncture, cranial trauma, or vascular anomaly, was found through extensive blood and imaging diagnostic workup. Although CAA is associated with increased risk of spontaneous SDH [6], our patient’s isolated hemorrhagic lesion, deep location, and lack of lobar involvement made CAA an unlikely primary factor.

As observed in a study, post-IPH, a proportion of patients developed intracranial hypotension [7]. One potential mechanism may involve altered ICP dynamics during hematoma resolution. As the IPH resolves and the surrounding cerebral edema subsides, it transiently lowers the ICP, theoretically placing stress on bridging veins [8]. If this stress exceeds the integrity of the venous wall, bilateral SDH formation may follow, especially in older adults with age-related vascular fragility. However, no clinical or radiological features of intracranial hypotension, such as orthostatic headache or brain sagging, were present in our patient. Thus, while low ICP remains a theoretical possibility, it cannot be substantiated in this case without objective evidence such as a lumbar puncture, which could not be performed in our patient.

The progression of the right-sided SDH without accompanying neurological symptoms is noteworthy. The non-contrast CT scan at week 8 showed predominantly isodense SDH with few areas of hyperintensity, and at week 14, predominantly hypodense SDH with areas of hyperintensity, depicting a sequential formation of SDH over the last two months with interval hemorrhage. This timing is crucial as it correlates with the resolution phase of IPH, while the 14-week scan shows near-complete resolution of primary IPH.

## Conclusions

Taking everything into account and having ruled out all the other possible causes of SDH, we suspect that the resolving IPH may be a risk factor in the development of SDH. Further research is required regarding the complexity of ICP dynamics in the resolution phase of IPH and the role it may play in the development of ICP. Additionally, we highlight the importance of an MDT approach with the appropriate specialist teams and therapy teams in improving overall patient outcomes, as evidenced in our patient. 
